# RNAseq Analysis Reveals Altered Expression of Key Ion Transporters Causing Differential Uptake of Selective Ions in Canola (*Brassica napus* L.) Grown under NaCl Stress

**DOI:** 10.3390/plants9070891

**Published:** 2020-07-14

**Authors:** Mobina Ulfat, Habib-ur-Rehman Athar, Zaheerud-din Khan, Hazem M. Kalaji

**Affiliations:** 1 Department of Botany, Government College University, Lahore 54000, Pakistan; zaheeruddin@gcu.edu.pk; 2 Department of Botany, Lahore College for Women University, Lahore 54000, Pakistan; 3 Institute of Pure and Applied Biology, Bhauddin Zakria University, Multan 66000, Pakistan; 4 Department of Plant Physiology, Institute of Biology, Warsaw University of Life Sciences SGGW, Nowoursynowska 159, 02-776 Warsaw, Poland; hazem@kalaji.pl

**Keywords:** RNAseq, transcriptome, *Brassica napus*, DEGs

## Abstract

Salinity is one of the major abiotic stresses prevailing throughout the world that severely limits crop establishment and production. Every crop has an intra-specific genetic variation that enables it to cope with variable environmental conditions. Hence, this genetic variability is a good tool to exploit germplasms in salt-affected areas. Further, the selected cultivars can be effectively used by plant breeders and molecular biologists for the improvement of salinity tolerance. In the present study, it was planned to identify differential expression of genes associated with selective uptake of different ions under salt stress in selected salt-tolerant canola (*Brassica napus* L.) cultivar. For the purpose, an experiment was carried out to evaluate the growth response of different salt-sensitive and salt-tolerant canola cultivars. Plants were subjected to 200 mM NaCl stress. Canola cultivars—Faisal Canola, DGL, Dunkled, and CON-II—had higher growth than in cvs Cyclone, Ac-EXcel, Legend, and Oscar. Salt-tolerant cultivars were better able to maintain plant water status probably through osmotic adjustment as compared to salt-sensitive cultivars. Although salt stress increased shoot Na^+^ and shoot Cl^−^ contents in all canola cultivars, salt-tolerant cultivars had a lower accumulation of these toxic nutrients. Similarly, salt stress reduced shoot K^+^ and Ca^2+^ contents in all canola cultivars, while salt-tolerant cultivars had a higher accumulation of K^+^ and Ca^2+^ in leaves, thereby having greater shoot K^+^/Na^+^ and Ca^2+^/Na^+^ ratios. Nutrient utilization efficiency decreased significantly in all canola cultivars due to the imposition of salt stress; however, it was greater in salt-tolerant cultivars—Faisal Canola, DGL, and Dunkled. Among four salt-tolerant canola cultivars, cv Dunkled was maximal in physiological attributes, and thus differentially expressed genes (DEGs) were assessed in it by RNA-seq analysis using next-generation sequencing (NGS) techniques. The differentially expressed genes (DEG) in cv Dunkled under salt stress were found to be involved in the regulation of ionic concentration, photosynthesis, antioxidants, and hormonal metabolism. However, the most prominent upregulated DEGs included Na/K transporter, HKT1, potassium transporter, potassium channel, chloride channel, cation exchanger, Ca channel. The RNA-seq data were validated through qRT-PCR. It was thus concluded that genes related to the regulation of ionic concentrate are significantly upregulated and expressed under salt stress, in the cultivar Dunkled.

## 1. Introduction

Salinity stress negatively affects the plant growth and yield of all glycophytic plants and cause multi-million dollar losses worldwide [1]. Growth reduction in plants occurs in salt-affected soil due to osmotic and ion toxic effects and limited uptake of nutrients [2]. However, due to the unique genetic makeup of every plant species, the extent of inhibitory effects varies greatly in all plant species, and some of them can cope with this problem better as compared to others. Canola (*Brassica napus* L.) has been reported to have the potential to cope with the toxicity of excess salt in the soil, and hence it can be successfully grown on salt-affected areas [3]. In an earlier study, screening was carried out for salt stress tolerance of 34 accessions of *B. napus,* and the selection of physiological parameters was done. Cultivar Dunkled was ranked salt stress-tolerant [4]. *Brassica napus* L. (Canola) ranks second after soybean, among oilseed crops, and provides 13% of the total oil supply in the world. However, further crop improvement up to 60% using current approaches by 2050 in this potential oilseed crop seems to be difficult. Thus, some novel sources of salt tolerance in this potential oilseed crop need to be explored. However, the problem is a lack of understanding of the detailed mechanism of salt tolerance or exact cellular and metabolic sites of salt-induced damages [5,6,7]. Several scientists have suggested that understanding how plants sense salt stress and respond to it through the various physiological processes will help us in devising a strategy to develop salt-tolerant plants [8]. For example, when plants are exposed to high salinity stress, membrane proteins at root hairs or root epidermal cells sense the extent of salt stress and communicate to whole plant body and alter different biochemical pathways, resulting in adjustments in ion homeostasis, detoxification of reactive oxygen species, changes in CO_2_ assimilation hormones, and growth regulation [9,10]. All these changes in the physiological process in response to salt stress are governed by the expression of salt stress-responsive genes or trigger the expression of some other related genes [11]. Salt overly sensitive (SOS) pathway is known to control ionic homeostasis in association with calcium-binding protein sensors that are important in signal transduction under salt stress [12,13,14]. The K^+^/Na^+^ balance under salt stress is also decisive in studying growth responses of plants under stress tolerance, and several transporters and their genes have been identified [14]. Serine/threonine type protein kinase CIPK24 is observed to be interacting with the Ca^2+^ sensor CBL4 and regulates the Na^+^/H^+^ exchangers and deliberates salinity tolerance [12]. These reports have suggested that genes can be grouped in sensing and signaling, ion transporters, and salt stress-related gene and transcriptional regulators. Identification of these genes playing their role in salt tolerance is necessary to develop salt-tolerant crops either through breeding or genetic engineering techniques.

In recent years, physiological processes complemented with proteome profiling and/or transcriptome mapping in plants have gained popularity to explore mechanisms of salt tolerance [15,16,17,18]. Next-generation sequencing (NGS) has become a potential technique to evaluate molecular profiles for crop plants [19]. It is being applied successfully throughout the world to identify transcriptome variation in plants [17,20]. Few studies on *B. napus* are available for the identification of salt-tolerant genes using the RNA-seq approach [16]. Keeping in view the above-mentioned facts, the first part of the present study was planned to evaluate salt tolerance in canola (*Brassica napus* L.) cultivars differing in salinity tolerance using the physiological and biochemical approach and to reconfirm behavior of cultivar Dunkled. In the second part, the transcriptome analysis (RNA-seq approach) was carried out in selected salt-tolerant cultivar Dunkled to identify key pathways and genes responsible for salt tolerance. Transcriptome analysis was further validated through gene expression of selected genes/salinity responsive transcripts. Our findings would help plant breeders and molecular biologists to improve cultivated canola varieties for better use of salt-affected areas all over the world.

## 2. Results

### 2.1. Evaluation of Different Cultivars of Canola (B. napus L.) for Salt Stress Tolerance

#### 2.1.1. Growth Attributes

**Figure 1:**
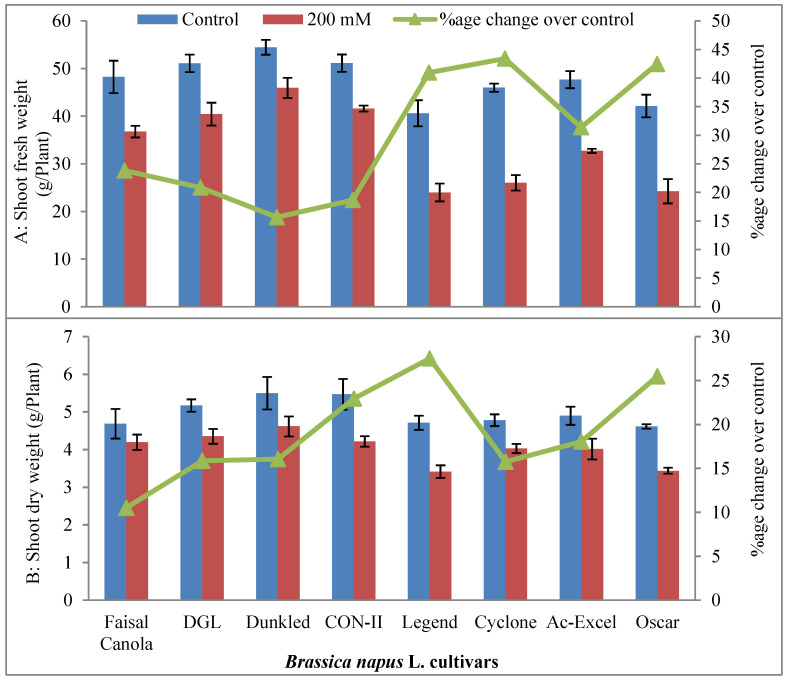
Shoot fresh (**A**) and dry weights (**B**) (g/plant) of eight cultivars of canola (*Brassica napus* L.) when two weeks old plants were grown under control (0 mM NaCl) or saline (200 mM NaCl) conditions for further five weeks (*n* = 3); Error bars are representing standard error.

Analysis of variance of the data for shoot fresh and dry weights of eight canola cultivars differing in salinity tolerance revealed that the imposition of salt stress caused a significant reduction in shoot fresh and dry weight (Table 1). Canola cultivars were significantly different in their fresh and dry weights under normal or saline (200 mM NaCl) conditions (Figure 1). As expected, salt-tolerant canola cultivars (DGL, Dunkled, Faisal Canola, and CON-II) had greater fresh and dry weights than those of salt-sensitive canola cultivars (Legend, Oscar, Cyclone, and Ac-Excel). However, Dunkled had maximum growth potential under saline conditions. However, cultivars—Legend and Oscar—were highly salt-sensitive being lowest in their fresh and dry weights of shoots.

#### 2.1.2. Relative Water Contents (RWC %)

Data for shoot dry weights of all canola cultivars under the study showed that the imposition of salt stress caused a significant reduction in shoot dry weights, as was evident from the analysis of variance (Table 1). Maximum dry weights under control conditions were observed in Dunkled (5.50 g), while minimum in Legend and Oscar. Under the influence of the saline environment, maximum dry weight was produced in Faisal Canola, DGL, Dunkled, and CON-II (4.19, 4.35, 4.61, and 4.21 g/plant, respectively) and statistically ranked same as the apparent differences had no statistical significance. Legend and Oscar were at the bottom end (Figure 1).

#### 2.1.3. Osmotic Potential (−MPa)

**Figure 2:**
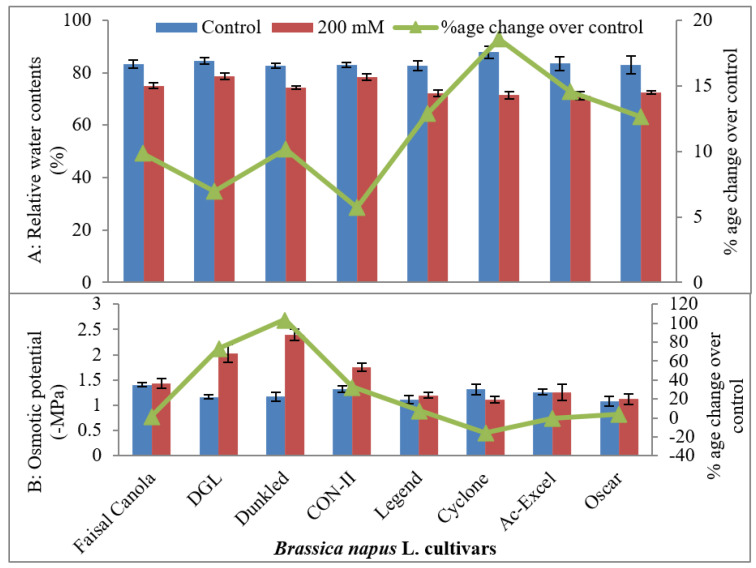
Relative water contents (**A**) (%) and osmotic potential (**B**) (−MPa) of eight cultivars of canola (*Brassica napus* L.) when two weeks old plants were grown under control (0 mM NaCl) or saline (200 mM NaCl) conditions for further five weeks (*n* = 3): Error bars are representing standard error.

Leaf osmotic potential decreased in the plants of all canola cultivars when grown under saline condition (Table 1; Figure 2). A maximum decrease in leaf osmotic potential was found in salt-tolerant cv Dunkled, whereas the lowest decrease in leaf osmotic potential (more negative) was observed in salt-sensitive canola cultivars—Legend, Cyclone, AC-Excel, and Oscar.

#### 2.1.4. Leaf Mineral Nutrients (Leaf Na^+^, K^+^, Ca^2+^, Cl^−^, K^+^/Na^+^ Ratio, Ca^2+^/Na^+^ Ratio)

**Figure 3:**
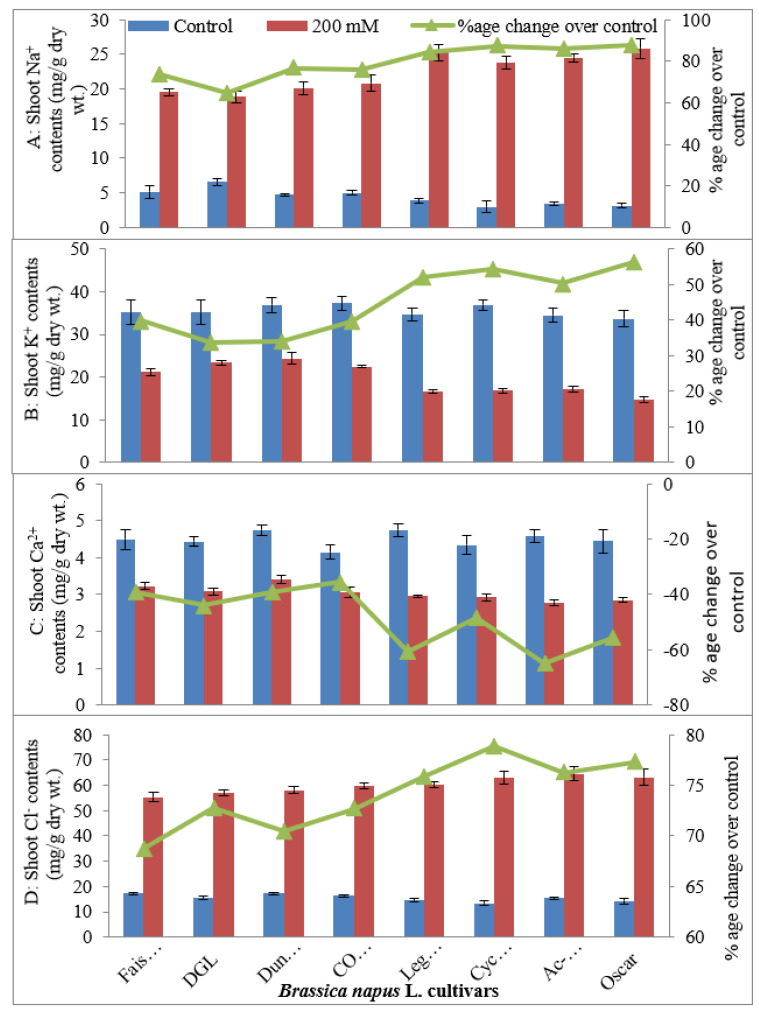
Shoot Na^+^ (**A**), K^+^ (**B**), Ca^2+^ (**C**), and Cl^−^ (**D**) contents (mg/g dry wt.) of eight cultivars of canola (*Brassica napus* L.) when two weeks old plants were grown under control (0 mM NaCl) or saline (200 mM NaCl) conditions for further five weeks (*n* = 3); Error bars are representing standard error.

Leaf Na^+^ and leaf Cl^−^ markedly increased in salt-stressed plants of all canola cultivars, whereas the accumulation of K^+^ and Ca^2+^ in the leaves decreased significantly due to the imposition of salt stress (Table 1; Figure 3). Moreover, the accumulation of Na^+^ in the leaves was lower in all four salt-tolerant cultivars than in all four salt-sensitive canola cultivars. Similarly, salt-tolerant canola cultivars had greater leaf K^+^ under saline conditions. A maximum increase in leaf K^+^ was observed in DGL and Dunkled, and minimum K^+^ uptake was observed in the cultivar Oscar (Figure 3). Salt-tolerant cv. Dunkled had significantly greater shoot Ca^2+^ contents under saline conditions as compared to those of all other cultivars (Figure 3). Whereas, salt-sensitive cultivars—AC-Excel and Oscar—showed minimum shoot Ca^2+^ contents. Maximum shoot Cl^−^ contents were observed in salt-sensitive cultivars—Legend, Cyclone, AC-Excel, and Oscar (Figure 3). In contrast, the minimum increase in shoot Cl^−^ contents was recorded in salt-sensitive cultivar Faisal Canola under salt stress conditions.

#### 2.1.5. Shoot K^+^/Na^+^ and Ca^2+^/Na^+^ Ratio

**Figure 4:**
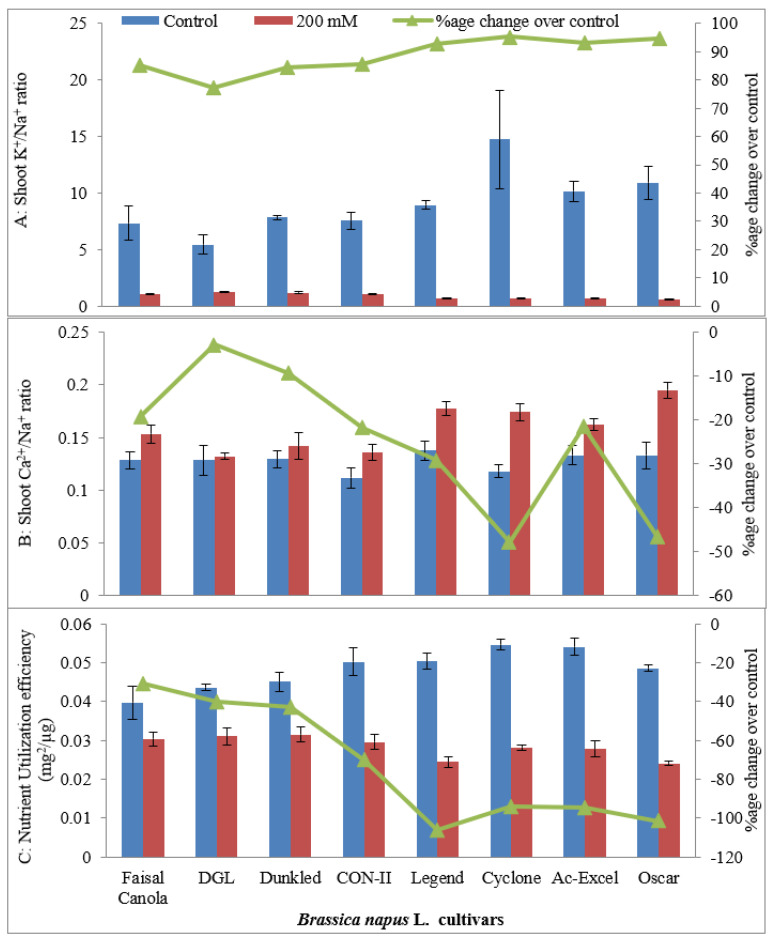
Shoot K^+^/Na^+^ ratio (**A**), Ca^2+^/Na^+^ ratio (**B**), and nutrient utilization efficiency (**C**) (mg^2^/µg) of eight cultivars of canola (*Brassica napus* L.) when two weeks old plants were grown under control (0 mM NaCl) or saline (200 mM NaCl) conditions for further five weeks (*n* = 3); Error bars are representing standard error.

Salt stress decreased the shoot K^+^/Na^+^ ratio in all canola cultivars (Table 1). The minimum K^+^/Na^+^ ratio was observed in salt-sensitive cultivar Oscar, while the highest K^+^/Na^+^ ratio was observed in salt-tolerant cultivar Dunkled. In contrast, shoot Ca^2+^/Na^+^ ratio remained the same in salt-tolerant cultivars, whereas it increased in salt-stressed plants of salt-sensitive cultivars of canola (Table 1; Figure 4).

#### 2.1.6. Nutrient Utilization Efficiency (mg^2^/µg)

It was evident from the statistical analysis of data regarding nutrient utilization efficiency that the imposition of salt stress in the rooting medium had a significant effect on nutrient utilization efficiency of canola cultivars (Table 1). Nutrient utilization efficiency decreased significantly in all the cultivars on the imposition of salt stress in the rooting medium (Figure 4). Under saline conditions, maximum nutrient utilization efficiency was observed in cultivars DGL and Dunkled. However, under saline conditions, minimum nutrient utilization efficiency was recorded in cultivar Oscar.

### 2.2. RNASeq Analysis and Differential Expression of Genes in Salt-Tolerant Cultivar Dunkled

On average, the control sample produced 58.97 million (58,969,962) reads, and total read bases were 8.9 G bp. The GC content was 49.15%, and Q30 was 94.06%. For the stress sample, 45.04 million (45,035,200) reads were produced with a total read base of 6.8 G bp. The GC content was 47.87%, and Q30 was 93.99%. Box plot showing raw expression (log_2_) is presented in (Figure S1). For the construction of contigs, raw reads were processed, as described in the Materials and Methods section. A total of 76,181 gene IDs were mapped on the reference genome (Table S1a). Differentially expressed genes (DEGs) were analyzed using R software. The upregulated genes were observed to be 29,187, and salt-stressed-induced downregulated genes were observed to be 29,291 (Table S1b). The analysis using DAVID revealed 17,912 characterized upregulated DEGs in control, and 20,931 characterized downregulated DEGs in the saline sample. Significant DEGs included sodium hydrogen exchanger (NHX), sodium transporter (HKT), potassium transporter (POT), Na-K-Cl co-transporter (NCKK1), cyclic nucleotide-gated ion channel 1 (CNGC1), mechanosensitive ion channel (MSL), potassium channel (KOR), chloride channel (ClCa), calmodulin (Calm), calmodulin binding transcriptional activator (CBTA), calcium transporting ATPase (Ca-ATPase), vacuolar ATPase (V-ATPase), heat shock proteins (HSP), late embryogenesis abundant proteins (LEA), Fe-superoxide dismutase (Fe-SOD), Cu-superoxide dismutase (Cu-SOD), Mn-superoxide dismutase (Mn-SOD), catalase (CAT), peroxidase (POD), tonoplast intrinsic protein (TIP), plasma-membrane intrinsic protein (PIP), nucleoplasm intrinsic protein (NIP), expansins (EXP), cell wall integrity and stress response component (WSC), NAC domain containing transcription factor (*NAC*), ethylene responsive transcription factor (*ERF*), MYB domain containing transcription factor (*MYB*), bZIP transcription factor (*bZIP*), 1-aminocyclopropane-1-carboxylic acid transcription factor (*ACC*), heat shock transcription factor (*HSP*) (Table S2a,b).

**Figure 5:**
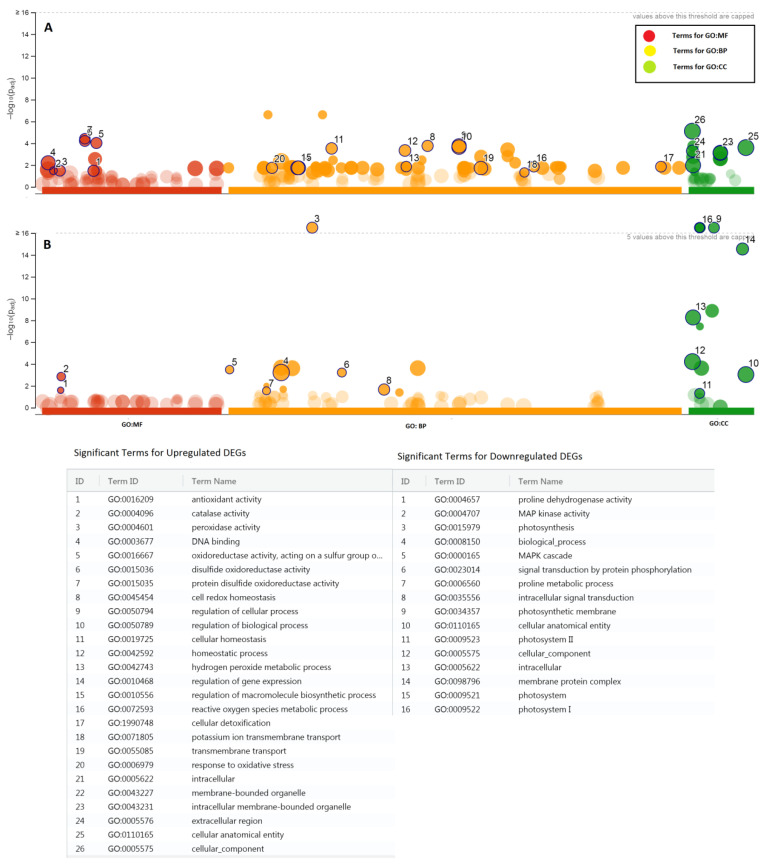
Bubble plot to represent functional profiling of significant gene ontology (GO) terms of selective upregulated (**A**) and downregulated (**B**) differentially expressed genes (DEGs) in canola (*Brassica napus* L.) grown under salt stress (Benjamini-Hochberg FDR threshold 0.05) using g:Profiler (version e99_eg46_p14_f929183). The x-axis represents z-score, whereas the y-axis represents the negative logarithm of the adjusted *p*-value. The area of the circles represents the number of genes assigned to the particular term. Detailed results are presented in Table S3a,b.

After selecting DEGs with fold change >5 and −5 and excluding DEGs other than protein-coding, DEGs were selected for gene function enrichment analysis by g:Profiler (https://biit.cs.ut.ee/gprofiler/gost). A bubble plot of statistically significant enriched terms is presented in Figure 5.

For upregulated DEGs, significant GO terms of the “Molecular Function” category included antioxidant activity (GO:0016209), DNA binding (GO:0003677), and peroxidase activity (GO:0004601). Significant GO terms of the “Biological Process” category included regulation of cellular process (GO:0050794), regulation of biological process (GO:0050789), homeostatic process (GO:0042592), hydrogen peroxide metabolic process (GO:0042743), cellular detoxification (GO:1990748), regulation of gene expression (GO:0010468), response to oxidative stress (GO:0006979), transmembrane transport (GO:0055085), and potassium ion transmembrane transport (GO:0071805). Significant GO terms of the “Cellular Component” category included intracellular membrane-bounded organelle (GO:0043231), cellular anatomical entity (GO:0110165), and cellular component (GO:0005575) (Table 2). For downregulated DEGs, significant GO terms of the molecular function category included MAP kinase activity (GO:0004707) and proline dehydrogenase activity (GO:0004657). Significant GO terms of the “Biological Process” category included MAPK cascade (GO:0000165), intracellular signal transduction (GO:0035556), and proline metabolic process (GO:0006560). Significant GO terms of the cellular component category included photosynthetic membrane (GO:0034357) and membrane protein complex (GO:0098796) (Figure 5).

Annotation by BlastKOALA resulted in 30 distinct terms with unique K numbers that were related to various transporter proteins, channels, and pumps (Table 2). It was inferred from these findings that salt stress caused over-expressed membrane-bound specialized proteins causing uptake of Na^+^, K^+^, Ca^2+^, and Cl^−^ ions, signal transduction, and triggering of differential gene expression by activation of various transcription factors (Table S4).

### 2.3. qRT-PCR

**Figure 6:**
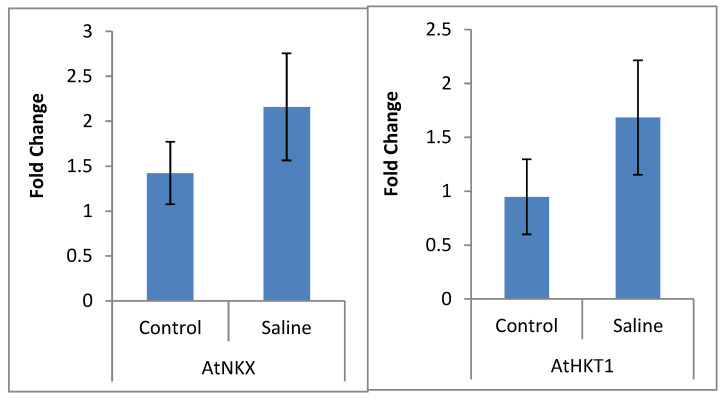
Expression (fold change) as depicted by qRT-PCR for two DEGs in leaf tissue of canola (*Brassica napus* L.) salt-tolerant cultivar Dunkled grown under saline (200 mM NaCl) conditions. Primer sequences are given in Table S5.

Transcriptome data was validated through qRT-PCR analysis. Among the upregulated genes, homologous genes of AtNKX and AtHKT1 were observed to be over-expressed in salt-stressed Dunkled plants when exposed to 200 mM NaCl (Figure 6).

## 3. Discussion

Plant growth responses to salt stress differ widely among glycophytes, reflecting differences in their genetic make-ups and respective potentials to withstand various stress levels [10]. Canola (*Brassica napus* L.) is moderately salt-tolerant with wide interspecific variation [1,21]. In the first part of the present study, four salt-tolerant and four salt-sensitive canola cultivars were used. Vegetative growth and development are some of the potential determinants of salt tolerance [22]. Plant growth results verified the degree of salt tolerance in canola cultivars examined in the present study. The existence of genetic diversity for abiotic stress tolerance in any crop species is prime for developing stress-tolerant cultivars through breeding programs [3]. Thus, studying physiological and biochemical responses in cultivars with diverse genetic background help in understanding the detailed mechanism of stress tolerance as well [10]. Although various physiological and biochemical processes are involved in salt stress tolerance, their contribution varies with species and type of cultivar. The well-known mechanisms can be categorized as osmotic tolerance, ion exclusion, and tissue tolerance [3,10,23,24]. However, several researchers have suggested that physiological traits contributing to these mechanisms should be evaluated individually as the ability of plants to maintain them under saline conditions [6,25,26,27]. Osmoregulation is an important phenomenon that is essential for normal cellular metabolism [28]. Under saline conditions, plants can accumulate organic and/or inorganic solutes, e.g., Glycine betain, proline, soluble sugars, Na^+^, K^+^, etc., to decrease its leaf osmotic potential, thereby increasing leaf turgor potential. Thus, if we establish a relationship between leaf relative water contents and osmotic potential, it becomes clear that leaf osmotic potential decreases with the decrease in relative water contents in plants grown under salt stress. Hence, an increase in turgor pressure is associated with a decrease in osmotic potential [29]. However, turgor by itself has no direct control on plant growth, rather it is essential for plant growth, as it is employed as an extending force required for the expansion of the cell wall [26]. In the present study, the leaf osmotic potential of all the cultivars decreased under the influence of saline conditions in the rooting medium. In plants grown in saline solution, lowest osmotic potential (more negative) was observed in Dunkled, while the highest was observed in Legend, Cyclone, AC-Excel, and Oscar. It is suggested that salt-tolerant canola cultivars maintained their water status through osmotic adjustment (either through the accumulation of organic solutes or inorganic osmotica) [26,30,31]. Leaf relative water contents of all the cultivars decreased under salinity stress. There was a marked increase in shoot Na^+^ and a decrease in shoot K^+^ contents in all canola cultivars under study, which resulted in a decrease in shoot K^+^/Na^+^ ratio. These results suggested that the accumulation of Na^+^ or K^+^ was not the main contributor to osmotic adjustment [24]. However, the K^+^/Na^+^ ratio was observed to show a positive relationship with salt tolerance, suggesting maintenance of ion homeostasis is more important in this regard [7].

Selection for salt-tolerant cultivars or identification of the mechanism of salt tolerance has been carried out through a variety of approaches, such as metabolite analysis, the gene for these metabolites through transcriptome analysis, miRNAs regulating genes responsible for metabolites and metabolic pathways, proteins responsible for specific pathways, or metabolites through proteome analysis, etc. [32,33,34,35]. Transcriptome studies to explore the mechanism of salt tolerance is gaining significant ground [16,17,34]. Thus, leaf transcriptome of salt-tolerant canola cultivar Dunkled was analyzed. Though roots are directly damaged by salt stress, growth inhibition of leaves in salt stress plants is more than that of roots. In the present study, differentially expressed genes (DEGs) were mainly involved in the regulation of ionic concentration in salt-tolerant cultivar Dunkled. Potassium transporters and channels upregulated under saline conditions, including two HKT1, two potassium transporter 9-like, one potassium transporter 4-like, one potassium channel KOR1 like, and one sodium/potassium/calcium exchanger. Greater salt tolerance in cv Dunkled was rendered to upregulation of transport proteins associated with sodium exclusion and preferential uptake of potassium. It was mediated either through adjusting K^+^ homeostasis by inducing the expression of potassium transporters or through vacuolar sodium sequestration by NHX, which is supported by the fact that salt stress upregulates vacuolar ATPases [34]. High Na^+^ concentration is reported to block high-affinity K^+^ transporters that result in reduced K^+^ influx and elevated Na^+^ influx [7,14,34].

Increased uptake of Ca^2+^ in the shoot was also reported to occur under salt stress, as was earlier observed in barley [36]. Nine calcium-sensing proteins were also upregulated, including calcium-sensing receptor chloroplastic like, calmodulin-like, and calmodulin-binding transcription activator proteins. Various reports have suggested that calcium sensory proteins activate sodium exclusion by activating plasma membrane or vacuolar Na^+^/H^+^ antiporters [14,24]. Downstream to calcium signaling MAP kinases may transduce the salt-induced osmotic stress signal and activate transcription factors by binding calmodulin and calmodulin-binding transcriptional activators [37]. Thus, the DEGs, like NHX, HKT, POT, NCKK1, CNGC1, MSL, KOR, ClCa, Calm, CBTA, Ca-ATPase, V-ATPase, HSPs, LEA, Fe-SOD, Cu-SOD, Mn-SOD, CAT, POD, TIP, PIP, NIP, EXP, WSC, *NAC*, *ERF*, *MYB*, *bZIP*, *ACC*, *HSP,* were inferred. Over-expression of AtNKX and AtHKT1 using qRT-PCR in Dunkled under salt stress validated that sodium exclusion and maintenance of potassium in leaf were mainly regulated through selective channels and transporters.

## 4. Materials and Methods

### 4.1. Evaluation of Different Cultivars of Canola (B. napus L.) for Salt Stress Tolerance

Canola (*Brassica napus* L.) plants from four salt-tolerant cultivars and four salt-sensitive cultivars were grown (25 plants/pot) in pots filled with sand. The sand was air-dried for 72 h, cleaned from straws, stones, and passed through 5 mm sieve in order to have uniformity. Pots were filled with 4.75 kg of sand. Thinning was done 5 days after completion of germination, leaving 6 plants/pot. Two-week-old plants were divided into two treatments, i.e., control (0 mM NaCl) and saline (200 mM NaCl). The salt solution was applied to target plant pots in such a quantity that the solution being eluted from the pot had the same EC (Electrical conductivity) as that of the irrigated salt solution, in order to attain a reliable stress level. Plants’ responses were observed and recorded for growth and physiological parameters after 5 weeks from the application of stress.

#### 4.1.1. Growth Attributes

Plants were harvested after five weeks of salt stress and separated into shoots and roots. Fresh weights of shoots and roots were recorded. In addition, shoot and root lengths were also recorded.

#### 4.1.2. Relative Water Contents (%)

Fully expanded youngest leaf (mostly 3rd leaf from the top) was cut from each replicate (one leaf per plant). Leaves were numbered, weighed, and the respective fresh weights were recorded. Leaves were transferred to a tray filled with water and kept for 60 min. Afterward, leaves were taken out, surface blotted, and weighed for turgid weights. Leaves were then kept in the oven for seven days at 65 °C, and dry weights were noted. Relative water contents were measured as follows:

(1) $$RWC=\frac{\left(Fresh\;weight-Dry\;weight\right)\times 100}{\left(Turgid\;weight-Dry\;weight\right)}$$

#### 4.1.3. Leaf Osmotic Potential (−MPa)

The third leaf from the top was excised from each replicate. Leaves were stored at −20 °C for a week. Frozen leaf material was allowed to thaw and thoroughly pressed with a glass rod to extract sap. The osmotic potential was determined using a vapor pressure osmometer.

#### 4.1.4. Mineral Contents (K^+^, Na^+^, Cl^−^, Ca^2+^)

The 0.1 g of powdered dry shoot material was digested using H_2_SO_4_ and H_2_O_2_, according to the method of [38]. The amount of Na^+^, K^+^, and Ca^2+^ ions in the digested shoots and roots were measured using a flame photometer (PFP 7, Jenway, UK). However, Cl^−^ ions were extracted in distilled water (10 mL). The mixture was continuously heated (80 °C) until the volume of the mixture was reduced to half. The volume of the mixture was restored to 10 mL with distilled water. Chloride meter was used to determine Cl^−^ contents in the samples.

#### 4.1.5. Nutrient Utilization Efficiency (mg^2^/µg)

Nutrient utilization efficiency was determined on the basis of shoot dry matter of the plant, following [39]:

(2) $$Nutrient\;utilization\;efficiency\;\left(\frac{mg^{2}}{µg}\right)=\frac{1\times shoot\;dry\;weight\;\left(mg\right)}{Shoot\;nutrient\;concentration\;\left(µg/mg\right)}$$

### 4.2. RNASeq Analysis and Differential Expression of Genes in Salt-Tolerant Cultivar Dunkled

#### 4.2.1. Isolation of Total RNA

Samples of leaves were harvested after 24 h of salt stress, immediately immersed in liquid nitrogen, and stored in a plastic zipper bag at −80 °C. Later on, leaves were ground in liquid nitrogen, and total RNA was isolated by an optimized protocol based on the hot borate method by Wan and Wilkins [40].

#### 4.2.2. Next-Generation Sequencing (NGS)

RNA was subjected to next-generation sequencing (Mcrogen, Seoul, Korea). Quality control (QC) was performed, and qualified samples proceeded to library construction. TruSeq Stranded mRNA LT Sample Prep Kit (Illumina, San Diego, CA, USA) was used using TruSeq stranded mRNA sample preparation guide, part # 15031047 Rev. E. The sequencing library was prepared by random fragmentation of the cDNA samples, followed by 5′ and 3′ adapter ligation. Alternatively, “tagmentation” combined the fragmentation and ligation reactions into a single step to increase the efficiency of the library preparation process. Adapter-ligated fragments were then PCR amplified and purified.

#### 4.2.3. Sequencing for Cluster Generation

The library was loaded into a flow cell where fragments were captured on a lawn of surface-bound oligos complementary to the library adapters. Each fragment was then amplified into distinct, clonal clusters through bridge amplification. When cluster generation was complete, the templates were ready for sequencing. Illumina SBS (sequencing by synthesis) technology utilized a proprietary reversible terminator-based method that detected single bases as they were incorporated into DNA template strands. As all 4 reversible, terminator-bound dNTPs were present during each sequencing cycle, natural competition minimized incorporation bias and greatly reduced raw error rates compared to other technologies. The result was highly accurate base-by-base sequencing that virtually eliminated sequence-context-specific errors, even within repetitive sequence regions and homopolymers.

#### 4.2.4. Generation of Raw Data

The Illumina sequencer generated raw images, utilizing sequencing control software for system control and base calling through integrated primary analysis software called RTA (real-time analysis). The BCL (base calls) binary was converted into FASTQ, utilizing Illumina package bcl2fastq.

#### 4.2.5. Bioinformatics Analysis of Raw Data

Data quality was assessed using the FastQC tool. Data contained “Nextera Transpose Sequence” adaptor content. Adaptor sequences were removed using fastp [41] software. High-quality reads (contigs) were assembled using TRINITY software. For functional annotation, assembled contigs were annotated using reference *Brassica napus* transcriptome data (https://www.ncbi.nlm.nih.gov/assembly/GCF_000686985.2/) through BWA [42] software (parameters: mem -t 4 -k 32 -M). Transcript counts were calculated using feature-count [43] software using -gene parameter.

#### 4.2.6. Bioinformatics Analysis of Feature Count Matrix

Differential expression of genes was determined using Bioconductor’s edgeR [44] package in R software (version: 3.5.3), yielding upregulated and downregulated DEGs. DEGs were converted to DAVID IDs online (https://david.ncifcrf.gov/conversion.jsp) using the gene ID conversion tool, selecting the official gene symbol as an identifier and *Brassica napus* as selected species. Only protein-coding DEGs were selected for further analysis. Selected DEGs with fold change (log2FC) greater than 5 (for upregulated DEGs) and −5 (for downregulated DEGs) were subjected to functional enrichment analysis of significant GO terms using g:Profiler (version e99_eg46_p14_f929183). Benjamini–Hochberg false discovery rate (FDR) threshold was set to 0.05. A bubble plot was made with the z-score (x-axis) against the negative logarithm of the adjusted *p*-value (−log P_adj_) (y-axis). Further annotation was carried out by KOALA (KEGG Orthology And Links Annotation) of KEGG GENES using BlastKOALA (https://www.kegg.jp/blastkoala/), and K numbers were assigned to the respective amino acid sequence data.

#### 4.2.7. Validation of NGS Data by qRT-PCR

The expression of two genes, i.e., AtNKX and AtHKT1, was assessed using RT-PCR. These genes were selected on the basis of having a documented role in salt tolerance mechanism [7,14,34]. RNA was converted to cDNA using Vivantis cDSK01-050 cDNA synthesis kit (Vivantis Technologies, 40170 Shah Alam, Selangor Darul Ehsan, Malaysia). Real-time polymerase chain reactions were performed on Mic PCR (Bio Molecular Systems, Brisbane Queensland, Australia). Beta-ACTIN was used as an internal control. Primer sequences are given in Table S5.

## 5. Conclusions

Salt tolerance in salt-tolerant canola cultivars—Faisal Canola, DGL, Dunkled, and CON-II—was found to be associated with maintenance of plant water status and salt exclusion. Salt-tolerant canola cultivars were better able to osmotically adjust and exclude toxic salts. Moreover, RNA-seq data verified that DEGs in salt-tolerant canola cultivar Dunkled were mainly related to membrane proteins related to water and ion transport (Na^+^, K^+^, Ca^2+^, Cl^−^).

It is thus concluded that salt stress upregulated various ion transporters while downregulated regulatory genes of signaling pathways. Among upregulated ion transporters, vacuolar and plasma membrane Na^+^/H^+^ antiporters, potassium transporters and channels (HKT1, KOR), calcium transporters, ABC transporters, cyclic nucleotide-gated ion channels (CNGCs), mechanosensitive ion channels, and chloride channels were included. Among the downregulated genes, transcripts related to MAPKs and senescence-associated genes were included. Therefore, enhanced salt stress tolerance observed in canola cultivar could be associated with water uptake, ion exclusion, and osmotic adjustment. The overexpression results of qRT-PCR with documented homologous *Arabidopsis thaliana* genes (AtNHX and AtHKT1) in salt-tolerant canola cultivar Dunkled further confirmed our findings.

## Figures and Tables

**Table 1:** Mean squares from the analysis of variance (ANOVA) of the data regarding eight cultivars of canola (*Brassica napus* L.) grown under control (0 mM NaCl) or saline (200 mM NaCl) conditions (*n* = 3).

Source of Variation	Cultivar (df = 7)	Salinity (df = 1)	Cultivar × Salinity (df = 7)	Error (df = 32)
**Shoot fresh weight**	257.58 ***	2254.50 ***	26.85 ^ns^	11.75
**Shoot dry weight**	0.79 **	10.71 ***	0.11 ^ns^	0.18
**Osmotic potential**	0.16 ***	1.35 ***	0.07 ***	0.01
**Relative water contents**	15.44 ^ns^	685.35 ***	32.28 *	10.42
**Shoot Na^+^ contents**	1.58 ^ns^	3579.38 ***	36.46 ***	6.12
**Shoot K^+^ contents**	209.51 ***	3291.80 ***	45.73 ***	7.57
**Shoot Ca^2+^ contents**	21.42 ***	643.15 ***	2.15 ^ns^	3.85
**Shoot Cl^−^ contents**	9.13 ^ns^	23919.01 ***	28.71 **	6.97
**Shoot Na^+^/K^+^ ratio**	0.18 ***	23.13 ***	0.10 *	0.03
**Shoot Ca^2+^/Na^+^ ratio**	0.01 ^ns^	8.46 ***	0.03 ^ns^	0.02
**Nutrient utilization efficiency**	3.12e−5 *	0.004 ***	7.53e−5 ***	1.28e−5

*** = significant at 0.001, ** = significant at 0.025, * = significant at 0.05, ^ns^ = non-significant. df = degree of freedom.

**Table 2:** KEGG (Kyoto Encyclopedia of Genes and Genomes) Orthology terms concluded from BlastKOALA for nucleotide sequences of the selective differentially expressed genes (DEGs) from plants of *B. napus* cultivar Dunkled grown under salt stress. (Detailed results are given in Table S4).

S.No	KEGG Orthology	K No.	Protein ID	Term Size
1.	calcium_sensing_receptor,_chloroplastic-like	K01013	XP_013677299	114
2.	calcium-transporting_ATPase_10,_plasma_membrane-type-like	K01537	XP_013668846	345
3.	V-type_proton_ATPase_subunit_H-like	K02144	XP_022557635	163
4.	V-type_proton_ATPase_subunit_G2	K02152	XP_013674837	79
5.	calmodulin-like_protein_12	K02183	XP_013736721	447
6.	potassium_transporter_4-like	K03549	XP_013689829	783
7.	mitogen-activated_protein_kinase_19	K04371	XP_013644611	603
8.	probable_cyclic_nucleotide-gated_ion_channel_14	K05391	XP_013660045	733
9.	ABC_transporter_B_family_member_13-like	K05658	XP_013650695	328
10.	ABC_transporter_C_family_member_7	K05666	XP_013668255	1477
11.	AP2-like_ethylene-responsive_transcription_factor_AIL1	K09285	XP_013746829	457
12.	ethylene-responsive_transcription_factor_ERF056-like	K09286	XP_022545196	151
13.	dehydration-responsive_element-binding_protein_2B-like	K09287	XP_022555207	394
14.	transcription_factor_MYB35-like	K09422	XP_013655211	310
15.	aquaporin_TIP3-1	K09873	XP_013649603	265
16.	aquaporin_NIP6-1_XP	K09874	013725889	305
17.	V-type_proton_ATPase_subunit_G3	K10604	XP_013669950	108
18.	mitogen-activated_protein_kinase_kinase_4-like	K13413	XP_013655549	353
19.	calmodulin-like_protein_8	K13448	XP_013710349	153
20.	sodium/potassium/calcium_exchanger_1-like	K13749	XP_013717164	288
21.	abscisic_acid_receptor_PYL10-like	K14496	XP_013741522	222
22.	mitogen-activated_protein_kinase_6-like	K14512	XP_013656013	392
23.	mitogen-activated_protein_kinase_1	K20535	XP_013640933	369
24.	mitogen-activated_protein_kinase_3	K20536	NP_001303218	370
25.	mitogen-activated_protein_kinase_7	K20537	NP_001303162	368
26.	mitogen-activated_protein_kinase_kinase_9	K20604	XP_013648942	308
27.	mitogen-activated_protein_kinase_kinase_kinase_ANP1-like_isoform_X1	K20606	XP_013641030	668
28.	mitogen-activated_protein_kinase_kinase_kinase_18-like	K20716	XP_013651099	456
29.	potassium_channel_KOR1-like	K21867	XP_022549779	632
30.	mechanosensitive_ion_channel_protein_9-like	K22048	XP_013659826	738

## Supplementary Materials

The following are available online at https://www.mdpi.com/2223-7747/9/7/891/s1, Figure S1: Box plot showing raw expression (Log_2_ scale) for control (0 mM NaCl) and saline (200 mM NaCl) plant samples from *Brassica napus* L. cultivar Dunkled, Table S1a: Gene count data for RNASeq from Canola (Brassica napus L.) plants grown under Control (0 mmol NaCl) or Saline (200 mmol NaCl) conditions, Table S1b: Fold Change (log2FC) for DEGs in Canola (Brassica napus L.) plants grown under Control (0 mmol NaCl) or Saline (200 mmol NaCl) conditions, Table S2a: List of selective upregulated DEGs (Fold change >5), Table S2b: List of selective downregulated DEGs (Fold change > −5), Table S3a: Functional enrichment analysis of selective upregulated DEGs (Fold change >5), Table S3b: Functional enrichment analysis of selective downregulated DEGs (Fold change > −5), Table S4: Annotation data for ion transporters after BlastKOALA, Table S5: List of primers used for qRT-PCR.
